# Development of physical activity policy and implementation strategies for early childhood education and care settings using the Delphi process

**DOI:** 10.1186/s12966-020-01034-2

**Published:** 2020-10-16

**Authors:** Hayley E. Christian, Donna Cross, Michael Rosenberg, Jasper Schipperijn, Trevor Shilton, Georgina Trapp, Stewart G. Trost, Andrea Nathan, Clover Maitland, Ashleigh Thornton, Elizabeth J. Wenden, Phoebe George

**Affiliations:** 1 Telethon Kids Institute, University of Western Australia, 35 Stirling Highway, Crawley, WA 6009 Australia; 2 School of Population and Global Health, University of Western Australia, Perth, Australia; 3 School of Human Sciences (Exercise and Sport Science), University of Western Australia, Perth, Australia; 4 Institute of Sports Science and Clinical Biomechanics, University of Southern Denmark, Odense, Denmark; 5 Institute of Health and Biomedical Innovation - Centre for Children’s Health Research, Queensland University of Technology, Brisbane, Australia; 6 Centre for Behavioural Research in Cancer, Cancer Council Victoria, Melbourne, Australia

**Keywords:** Physical activity, Policy, Intervention, Childcare, Delphi, Screen time, Sedentary behaviour, Guidelines, Pre-school

## Abstract

**Background:**

The aim of this study was to gain consensus on an evidence informed physical activity policy template for early childhood education and care (ECEC) and determine best-practice dissemination and implementation strategies using the Delphi process.

**Methods:**

Three-round modified Delphi methodology. During round one an expert working group developed an evidence informed ECEC specific physical activity policy template. Rounds two and three involved national online surveys to seek insight from a group of experts on the draft physical activity policy template.

**Results:**

Ninety per cent of experts reported ECEC services are fully responsible for having a physical activity policy. There was consensus on the components of the policy and key physical activity and sedentary behaviour statements and recommendations. The most effective methods for disseminating a physical activity policy to ECEC providers included online (websites, social and electronic media), ECEC targeted launch events, direct mail outs and via professional associations. Twenty five key strategies related to management, supervisors and educators; the ECEC physical environment; communicating with families; and accreditation, monitoring and review, were identified as necessary for the successful implementation of physical activity policy in ECEC. Experts reached consensus on nine of these strategies indicating they were both easy to implement and likely to have a high level of influence. Key barriers and enablers to implementing ECEC-specific physical activity were also identified.

**Conclusions:**

This evidence informed physical activity policy template for ECEC provides recommendations on the amount of physical activity and sedentary time (including screen time) children should have whilst attending ECEC and aligns with national/international guidelines. A number of effective physical activity policy implementation strategies for ECEC were identified. An important next step is advocating for the introduction of legislative requirements for services to have and implement a physical activity policy.

## Background

Daily physical activity is critical for a young child’s health and development [1, 2]. Being physically active helps young children build musculoskeletal and bone strength, develop their cardiorespiratory fitness, maintain a healthy weight and contributes to their social-emotional, cognitive and physical development [3]. Physical activity and sedentary behaviours can track from early childhood into adolescence and adulthood [4], either positively or negatively influencing health throughout the life course.

In conjunction with Canada, Australia released the first 24-h Movement Guidelines for the Early Years in 2017 [5]. The Guidelines include recommended levels of physical activity, sedentary behaviour and sleep in a 24-h period for infants (0–1 year), toddlers (1–2 years) and pre-schoolers (2–5 years) [6]. Similarly, in 2018 the World Health Organization, for the first time, released guidelines on physical activity, sedentary behaviour and sleep for children under 5 years of age [7]. These guidelines recommend that young children aged 2 to 5 years spend at least 3 h per day in a variety of physical activities, including energetic play, spread throughout the day, with more time being better [6]. Furthermore, pre-schoolers should not be confined for more than 60 min at a time, sit for extended periods and sedentary screen time should be less than 60 min per day [6]. While international public health guidelines emphasise the benefits of physical activity from a young age, many pre-schoolers do not meet physical activity recommendations [8, 9]. Device-based measures of physical activity show less than a third of Australian pre-schoolers achieve the recommended 3 h of physical activity per day required for health and development [10, 11].

Over a half of all 2–3-year-olds in Australia attend an early childhood education and care (ECEC) service [12]. ECEC services, such as long day care, are an important setting for increasing physical activity in the early years, yet international evidence shows that a significant proportion of pre-schoolers fail to meet physical activity recommendations whilst attending ECEC [13, 14]. This is also the case in Australia; less than one in 10 children attending ECEC meet the recommended 3 h of physical activity per day [10]. Internationally, some evidence shows that ECEC policies supportive of children’s physical activity result in higher levels of physical activity [15], yet only about a half of all Australian, New Zealand, Canadian and US services have a written physical activity policy [16–19]. There is also considerable variation within countries; for example, in Australia, 58% of services in New South Wales have a written physical activity policy [16] while only 16% of Western Australian services have one or more physical activity-related statements present in center policies [10]. Overall, there is a need for focused research to develop, implement and evaluate evidence informed physical activity policy specifically for ECEC and to measure its impact at the child, educator and organizational level. Importantly, strategies to facilitate implementation of physical activity policies in ECEC must consider the local implementation context and barriers (e.g., staff knowledge and preferences, lack of space, time, cost) [15].

ECEC is highly regulated in Australia. The Australian National Quality Framework for Early Childhood Education and Care was implemented in 2012 to provide minimum standards across seven Quality Areas [20]. Quality Area 2.1 states that ‘Each child’s health and physical activity is supported and promoted’. The national regulatory body’s (Australian Children’s Education & Care Quality Authority) 2019 annual report states that services find Quality Area 2.1 more challenging to meet than most other standards with the second lowest number of services rated as ‘Exceeding’ [21]. Overall, there is little guidance, resources or training available from the regulatory body or from other sources to enable service providers and educators to support and promote children’s physical activity at ECEC [22].

While ECEC physical activity guidelines exist in Australia (‘Healthy Eating and Physical Activity Guidelines for Early Childhood Settings’) [23] they are the same as the national guidelines and are not specific to the time children spend in care. Moreover, ECEC policy guides exist for other areas of early child health and development. For example, policies for children’s sun protection are widely adopted and successfully implemented in ECEC by the Australian Cancer Council [24, 25]. Given the level of children’s physical inactivity whilst attending ECEC and the absence of written policies specifying the amount of physical activity, it is important that policy be developed and implemented to promote young children’s physical activity whilst in care. The aim of this study was to develop an ECEC specific physical activity policy template and determine best-practice dissemination and implementation strategies using the Delphi process and expert consensus.

## Methods

### Research design

This project formed part of the PLAY Spaces and Environments for Children’s Physical Activity (PLAYCE) Policy Partnership Project to develop, implement and evaluate evidence informed physical activity policy for ECEC. We used a three-round Delphi methodology. The Delphi methodology is a repeated survey research process which implements responses from multiple relevant individuals to improve the quality of decision making [26]. It can be effectively used to develop, review, and refine policy [27, 28]. For round one we consulted the peer-review literature and worked with an expert group to develop an evidence informed ECEC specific physical activity policy template. A national and international sample of experts reviewed, provided feedback, and reached agreement on a first draft physical activity policy template (June–August 2019). Rounds two (September 2019) and three (November 2019) involved national online surveys to seek insight from a different group of experts on the draft physical activity policy template.

### Participants

The round one Delphi expert group meetings involved 10 national and international academics with expertise in children’s physical activity (research, advocacy, programs and policies) and early childhood development, and nine stakeholder representatives from ECEC service provision, ECEC professional associations, as well as government (health, sport and recreation) and non-government organizations (National Heart Foundation, Cancer Council, Nature Play Australia) focused on promoting children’s physical activity. Participants were a convenience sample selected to provide sufficient breadth and depth of relevant expertise with an average of at least 10 years’ experience in their field of work. Round one participants did not take part in subsequent rounds.

For the Delphi rounds two and three we used existing partnerships and collaborations to identify individuals with expertise across the areas of children’s physical activity research and programs, child health and development, the ECEC sector and policy development. Representatives from all major organisations working in the area of young children’s physical activity and or ECEC were identified. We sought representation across all Australian States and Territories. The round two online survey link was emailed and received by 243 experts of which 149 consented to participate (response rate 61%). The round three online survey link was emailed and received by 251 experts of which 89 consented to participate (response rate 35%; 75% completed the first survey). For both survey rounds, participants were sent a reminder email to complete the survey 1 week after the initial invitation email. An extra eight experts received the round three online survey email as a result of it being forwarded on to colleagues. All participants provided informed consent to participate. The anonymity of all participants was maintained throughout rounds two and three. The study was approved by The University of Western Australia’s Human Research Ethics Committee (#RA/4/20/5597).

### Data collection and analysis

#### Round one: expert input to draft an ECEC physical activity policy

The aim of round one was to use existing evidence and the expert group to develop a physical activity policy template for ECEC services. The expert group had monthly face-to-face meetings as well as email exchange over a six-month period. The policy structure was based on the widely adopted and successfully implemented Cancer Council ‘Sample SunSmart policy for ECEC services’ [25]. Key elements of the SunSmart policy for ECEC services included: a) Purpose and rationale for the policy; b) Relevent legislation and standards; and c) Procedures (strategies) for meeting the policy. The existing peer review literature was used to identify evidence-based strategies to include in the physical activity policy template for ECEC that would assist services with implementation. Implementation frameworks such as the Consolidated Framework for Implementation Research [29] were considered when identifying which of these strategies to include in the physical activity policy template. The expert group were asked to review and provide feedback on the policy structure, content, relevant legislation, strategies for implementation and policy monitoring and review. The expert group’s comments and edits were incorporated, into the physical activity policy template for ECEC services prior to each monthly face to face discussion. The goal was to reach consensus on the physical activity policy template so it could be used to seek broader input and feedback via a national Delphi survey.

#### Round two: content and coverage of draft physical activity policy for ECEC settings

The aim for the second round of the Delphi was to refine the content of the physical activity policy template for ECEC. Data were collected using an online Delphi survey, which included items assessing sociodemographic characteristics to capture participant representation across Australian States and Territories, urban/rural areas, different stakeholder groups and time employed in their sector. Participants were provided with definitions of ECEC, physical activity and sedentary behaviour and the Australian 24-h Movement Guidelines for the Early Years [6]. Participants were asked whether having a physical activity policy was a responsibility of ECEC services (response options: definitely, somewhat or not at all responsible); their level of agreement with key features (rationale, scope, key statements and recommendations, relevant legislation, procedures/strategies, supporting resources, monitoring and review, other) that should be included in an ECEC physical activity policy (5 point scale: strongly disagree; disagree; neither disagree/agree; agree; strongly agree); the level of acceptability of six key physical activity and sedentary behaviour statements and recommendations for infants, toddlers and pre-schoolers (5 point scale: highly unacceptable; unacceptable; neither; acceptable; highly acceptable), and to suggest wording changes for the key statements and recommendations. Participants also reported the acceptability (5 point scale: highly unacceptable; unacceptable; neither; acceptable; highly acceptable) and ease of implementation (5 point scale: very difficult; difficult; neither; easy; very easy) of 25 ECEC-specific physical activity policy implementation strategies, as well as suggested wording changes and new strategies and ranking of the three easiest and three hardest strategies to implement. Strategies were presented under four sub-groups: Management/Supervisors/Educators; Physical environment; Communicating with families; and Accreditation, monitoring and review. Participants reported how the policy could be best disseminated to ECEC services, ranked the top three dissemination strategies and identified the single most effective dissemination strategy. Finally, participants were asked if they were provided with a physical activity policy to implement in their/an ECEC, what would be the top three barriers and the top three enablers for: a) educators; b) the ECEC organisation; c) ECEC physical environment; and d) parents and the community. All Delphi survey questions included an option for open-ended responses and to make suggested wording changes.

Descriptive analyses were conducted. A total of 123 participants provided complete data to be included in round two analyses. Items with ≥70% agreement (i.e., participants responded agree or strongly agree; acceptable or highly acceptable; easy or very easy) were deemed to have consensus [30]. The wording of key physical activity and sedentary behaviour statements and recommendations were edited based on feedback.

#### Round three: key statement and recommendation consensus, implementation strategy prioritisation and identification of key barriers and enablers to implementation strategies

The aim of the third round of the Delphi process was to report results from the previous round to back to participants, gain consensus on the remaining key statements and recommendations and gain further input about implementation strategies level of influence and ease of implementation. Using an online survey, participants reported how easy it would be for a service to implement each strategy, if they had 1 year to implement the policy (5 point scale: very difficult; difficult; neither; easy; very easy), and the level of influence (i.e. the power a strategy has to be a barrier or enabler to successful policy implementation) it would have on overall implementation of the physical activity policy in ECEC services (5 point scale: very weak; weak, moderate; strong; very strong). Strategies were presented in the same sub-groups as round two’s survey: Management/Supervisors/Educators; Physical environment; Communicating with families; and Accreditation, monitoring and review. Finally, using the results from the first round survey and relevant systematic reviews [15, 31, 32] a list of key barriers and enablers to strategies were proposed. Participants then ranked the top five enablers and top five barriers to implementation strategies focused on Management/Supervisors/Educators; Physical environment; and Communicating with families.

Descriptive analyses were conducted. A total of 80 participants provided complete data to be included in round three analyses. Items with ≥70% agreement (i.e., participants responded easy or very easy; strong or very strong) were deemed to have consensus [30]. Cross-tabulations were used to identify consensus implementation strategies that were both easy to implement and reported as having a high level of influence on overall implementation of the physical activity policy in ECEC services.

## Results

### Sample characteristics

The round one expert group of 10 academics and nine stakeholder representatives all had a bachelor degree or higher, 42% were female, the majority were based in Western Australia (68%) with additional representation from Victoria, Queensland, nationally and internationally. All but one of the academics were experts in physical activity intervention research (the other in early child development and ECEC); the average number of publications in the last 5 years was 57. Of the stakeholders, there were two representatives from the ECEC sector, two from government (health and physical activity policy and programs) and five from non-government organisations (National Heart Foundation, Cancer Council, Nature Play Australia, Minderoo Foundation - CoLab for Kids). Most of the expert group (68%) had been employed in their field for more than 10 years.

The second-round survey (*n* = 123) showed representation from all Australian States and Territories with the largest response from Western Australia (63%). The majority of respondents (89%) were located in a metropolitan area compared with rural or remote areas. Different stakeholder groups were well represented including ECEC providers, physical activity related researchers and organisations with a remit to promote physical activity and government policy. The majority (87%) of respondents had been employed in their area for three or more years. Among stakeholder groups, most were involved with ECEC or the early years sector (37%) followed by research (28%) and then government (24%). Sixty percent of respondents had a bachelor’s degree or higher (Table 1).

**Table 1:** Characteristics of expert respondents in round two survey (*n* = 123)

	N (%)
Geographical location:	
Western Australia	78 (63.4)
Queensland	12 (9.8)
New South Wales	12 (9.8)
South Australia	8 (6.5)
Victoria	3 (2.4)
Australian Capital Territory	4 (3.2)
Tasmania	5 (4.1)
Northern Territory	1 (0.8)
Metropolitan	110 (89.4)
Rural	11 (9.0)
Remote	2 (1.6)
Stakeholder group:	
ECEC/early years sector	45 (36.6)
Researcher	35 (28.5)
Government	30 (24.4)
Non-government organisation	9 (7.3)
Other	4 (3.2)
Years employed in stakeholder group identify with:	
> 15	38 (30.9)
10–15	26 (21.1)
5–9	26 (21.1)
3–4	17 (13.9)
< 2	16 (13.0)
Highest level of education completed:	
Secondary school or less; Trade/apprenticeship	2 (1.6)
Certificate/Diploma 2 years or less	5 (4.1)
Certificate/Diploma 3 years or less	10 (8.1)
Bachelor degree or higher	74 (60.2)
Missing	32 (26.0)

### ECEC physical activity policy needs and features

Overall, 89% of respondents believed it was definitely the responsibility and 11% somewhat the responsibility of ECEC services to have a physical activity policy. Consensus was reached that an ECEC specific physical activity policy should include a rationale (92% agreement), scope (91% agreement), key statements (93% agreement), key recommendations (91% agreement), links to legislation (85% agreement), procedures for implementation (referred to hereon as strategies) (93% agreement), supporting resources (86% agreement) and policy monitoring and review (90% agreement) components.

### Acceptability of ECEC physical activity policy key statements and key recommendations

All seven of the ECEC policy physical activity and sedentary behaviour key statements and recommendations for infants, toddlers and pre-schoolers reached consensus that they were acceptable (see Table 2).

**Table 2:** Acceptability of ECEC policy physical activity and sedentary behaviour key statements and recommendations^a^

	% agree acceptable^**b**^
**Key statements**	
* Encourage physical activity in young children *Our service will provide infants, toddlers, and pre-schoolers with opportunities to be physically active throughout the day.*	92%
* Limit sedentary behaviours in young children *Our service is committed to using strategies to break up prolonged sitting and limiting the total amount of time young children spend sitting. This includes limiting the use of equipment that restricts movement*.	77%
**Key recommendations -** ***Toddlers & Pre-schoolers (1–5 years)***	
* At least 120–150 min (2–2 ½ hours) spent in a variety of physical activities, including energetic play, spread throughout the day*. More is better. (*Based on a standard 6–8 h day in care) *Children who spend 180 min in a variety of physical activities, including energetic play, spread throughout the day will meet the Australian 24-Hour Movement Guidelines for the Early Years.*	76%
ALTERNATIVE WORDING:	
* At least a third of the day should be spent in a variety of physical activities, including energetic play, spread throughout the day. More is better. *Children who spend 180 min in a variety of physical activities, including energetic play, spread throughout the day will meet the Australian 24-Hour Movement Guidelines for the Early Years.*	84%
* Toddlers and pre-schoolers should not be confined for more than 60 min at a time (e.g., in a stroller or highchair). Children should not sit for extended periods (except when engaged with a caregiver e.g., reading and storytelling). Less is better. Sedentary screen time for purposes other than learning should not be allowed.	88%
**Key recommendations –** ***Infants (under 1 year)***	
* Infants and babies to be physically active in a variety of ways, particularly through supervised interactive floor-based play, including crawling and games. More is better. For infants not yet mobile, provide at least 30 min of tummy time spread throughout the day which includes reaching and grasping, pushing and pulling.	96%
* Ensure cots, car seats, and high chairs are used for their primary purpose only (cots for sleeping, car seats for vehicle travel, and high chairs for eating). Limit the use of equipment such as strollers, swings, and bouncer seats/chairs for holding infants while they are awake. Screen time for infants is NOT recommended.	87%

^a^Round two survey wording presented

^b^Percentage of respondents answered acceptable or highly acceptable

### ECEC physical activity policy implementation strategies

There was consensus that all 25 strategies were acceptable to implement a physical activity policy in ECEC. There was consensus that each strategy would have a strong level of influence on the overall implementation of a physical activity policy in ECEC services, with the exception of two strategies related to communicating with families (i.e., Provide a copy of the physical activity policy to all families upon orientation at the service; and Families will be provided with opportunities to contribute to the review and development of the policy) (see Table 3). Eight of the total 25 strategies had 70% or more agreement across respondents that the strategies would be easy to implement over a one-year period. Seven out of the eight easy-to-implement strategies were also reported to have a strong level of influence on the overall implementation of a physical activity policy in ECEC: 1) Provide many daily opportunities for outdoor play time; 2) Program a range of learning experiences encouraging and using active play and for children to be physically active; 3) Break up prolonged periods of sedentary behaviours e.g. sitting or standing for long periods or infants being confined to high-chairs or cots if they are not eating or sleeping; 4) Not use punitive measures such as withholding physical activity as punishment for managing challenging behaviours (e.g. seated time out) and not use physical activity as punishment (e.g. star jumps); 5) Foster awareness and understanding of this physical activity policy; 6) Physical activity policy is available to staff, families and visitors; and 7) Wear comfortable and appropriate clothing and footwear that doesn’t limit children’s and educator’s ability to engage in physical activity.

**Table 3:** ECEC physical activity policy implementation strategies by ease of implementation and influence level

	Strong level of influence^**a**^	Easy to implement^**b**^	> 70% agree easy to implement AND strong level of influence
**Management/Supervisors/Educators**			
1. Foster awareness and understanding of this Physical Activity Policy	80%	83%	✓
2. Provide many daily opportunities for outdoor play time	91%	91%	✓
3. Embed the importance of active play and physical activity in everyday experiences	91%	69%	
4. Program a range of learning experiences encouraging and using active play and for children to be physically active	87%	73%	✓
5. Provide opportunities for children to engage in discovery learning and discussion on the importance of physical activity	78%	62%	
6. Break up prolonged periods of sedentary behaviours e.g. sitting or standing for long periods or infants being confined to high-chairs or cots if they are not eating or sleeping	87%	74%	✓
7. Limit the use of equipment such as strollers, swings and bouncer seats/chairs for holding infants while they are awake	83%	57%	
8. Not use punitive measures such as withholding physical activity as punishment for managing challenging behaviours (e.g. seated time out) and not use physical activity as punishment (e.g. star jumps)	80%	81%	✓
9. Include physical activity as part of the assessment of children’s physical and overall development	76%	56%	
10. Ensure age and developmentally appropriate structured and unstructured physical activity is provided for each child	96%	59%	
11. Act as positive role models and demonstrate and participate in active play and physical activity with children	85%	54%	
12. Take part in professional development programs to increase knowledge and skills around children’s physical activity	91%	52%	
13. Provide opportunities for all children (including children with disabilities) to be physically active. Children with disabilities should be provided with equipment that meets the current standards for accessible design to encourage physical activity	89%	37%	
**Physical environment**			
14. Wear comfortable and appropriate clothing and footwear that doesn’t limit children’s and educator’s ability to engage in physical activity	83%	72%	✓
15. Provide an outdoor environment with a variety of portable and fixed play equipment, a secure perimeter, shade, natural elements, open grassy areas, free running space, connected paths, varying surfaces and terrain, and more than the minimum outdoor space per child where possible	83%	30%	
16. Provide an indoor environment with a variety of portable (and fixed) play equipment, natural elements, free running space, and more than the minimum indoor space per child where possible	85%	34%	
17. Ensure adequate physical activity opportunities in poor weather such as very high or low temperatures, storms or UV index ratings above 8. Provide indoor physical activity alternatives where possible	85%	43%	
**Communicating with families**			
18. Provide a copy of the Physical Activity Policy to all families upon orientation at the service	60%	98%	
19. Talk with families about their children’s physical activity	77%	56%	
20. Communicate regularly with families about physical activity experiences within our service and provide information to assist families to support their child to have many opportunities to engage in active play and physical activity at home	74%	60%	
21. Encourage parents to support their child to have many active play and physical activity experiences at home	71%	44%	
22. Families will be provided with opportunities to contribute to the review and development of the policy	60%	53%	
**Accreditation, monitoring and review**			
23. Services will be able to have their policy reviewed and becomes an accredited physical activity promoting centre	83%	45%	
24. This Physical Activity Policy is available to staff, families and visitors	72%	100%	✓
25. All staff, including management, educators and parents, monitor and review the effectiveness of the policy and revise the policy when required (at least once every 3 years)	77%	60%	

^a^Percentage of respondents answered strong or very strong

^b^Percentage of respondents answered easy or very easy

### ECEC physical activity policy implementation strategy barriers and enablers

Key barriers to policy implementation strategies focused on Management/Supervisors/Educators were related to existing workloads and competing priorities, lack of training and/or professional development and costs in time, resources and money (Table 4). Key enablers to policy implementation strategies focused on Management/Supervisors/Educators were related to funding availability, a clear, concise and easy to follow policy document and legislative or National Quality Standard requirements. The amount of indoor and outdoor space as well as play equipment available were reported as key enablers and barriers to the implementation of strategies focused on Management/Supervisors/Educators and the ECEC Physical environment (enablers only).

**Table 4:** Key barriers and enablers to strategies for implementing a physical activity policy in ECEC

Implementation strategies focus	Barriers	Enablers
Management/ Supervisors/ Educators	Existing workload & competing priorities	
	Lack of training and/or professional development	
		Legislative or National Quality Standards requirement
		Clear, concise and easy-to-follow policy document
	Costs in time, resources and money	Funding available
	Lack of available indoor/outdoor space; Insufficient equipment	Indoor/outdoor space available
Physical environment	Cost of environmental upgrade or change	
	Parental perception of risk or injury/illness	
	ECEC/staff perception of injury or illness	
		Availability of indoor and outdoor space
		Portable & play equipment and resources available
		Access to local green spaces
	Weather extremes	Mitigating weather extremes
Communicating with families	Time poor parents	
	Parental lack of interest in physical activity	
	Parental expectation of service (e.g. school readiness)	
	Conflict with rules & norms at home (e.g. screen time)	
		Active parent engagement
		Supportive parent-educator relationships
		Ambassadors to promote & raise awareness

Parental and ECEC/staff perception of risk, injury or illness and the cost of environmental upgrades or changes were reported as key barriers to implementation strategies focussed on the ECEC physical environment. Weather extremes were considered both a key barrier and enabler (i.e., mitigating weather extremes) to ECEC physical environmental implementation strategies. Having access to local green space was reported as a key enabler.

Key barriers to implementation strategies focused on communicating with families included time poor parents, parental lack of interest in physical activity, parental expectations of the service (e.g., school readiness) and conflicts with rules and norms at home (e.g., around screen time). Enablers included supportive parent-educator relationships, active parent engagement and having local ambassadors to raise awareness and promote the physical activity policy.

### Best practice strategies for disseminating a physical activity policy to ECEC providers

The most effective strategies reported for disseminating a physical activity policy to ECEC providers were via: websites, social and electronic media; ECEC targeted launch events; direct mail outs (including hard copies of the policy); and through professional associations.

## Discussion

Almost 90% of experts reported it is the full responsibility of ECEC services to have a physical activity policy. There was also consensus on the components, as well as key physical activity and sedentary behaviour statements and recommendations that should be included in an ECEC specific physical activity policy. The key components of a comprehensive physical activity policy for ECEC included a rationale, scope of the policy, key statements and recommendations (on the amount of physical activity and sedentary time per day at ECEC), links to relevant legislation, procedures for implementation, supporting resources and policy monitoring and review. The findings also highlighted that ECEC physical activity policies should be evidence based and aligned with national and international (e.g., World Health Organization) 24-h movement guidelines [33].

In Australia, the National Quality Framework for Early Childhood Education and Care minimum standard Quality Area 2.1.3 states that ‘Healthy eating and physical activity are promoted and appropriate for each child’ [20]. However, there is little guidance, and few resources available (including physical activity policy templates) detailing how services go about promoting children’s physical activity. By consulting the literature and involving a range of stakeholders in developing the policy template we were able to ensure that it was evidence-based while still reaching consensus on the key physical activity and sedentary behaviour statements and recommendations included. An evidence informed physical activity policy template as developed in this study will provide clear guidance on the amount of physical activity and sedentary time (including screen time) children should have whilst attending ECEC and align with national/international guidelines.

The ECEC specific physical activity policy template was comprehensive with consensus reached on the inclusion of 25 acceptable strategies. Very few instances exist internationally where comprehensive and detailed physical activity strategies are included in ECEC regulations at a state or national level [34]. One example is the Alberta (Canada) Child Care Accreditation Standards which include nine child physical activity related indicators that cover promoting physical activity and minimising sedentary time, planning and programming for children’s physical activity, modelling and indoor and outdoor physical activities [35]. These indicators are similar to the management/supervisor/educator strategies included in the physical activity policy template. The strategies included in the policy template are more comprehensive for managers/supervisors and educators and uniquely capture strategies relating to the ECEC physical environment, parent engagement and policy monitoring.

The majority of studies to date report the impact of the presence only (not content) of ECEC physical activity policies on children’s physical activity, with many showing little effect due to poor implementation [15, 36]. However, it is likely that the content, comprehensiveness, acceptability, ease of use and implementation strategies included in ECEC physical activity policies are also important, rather than the mere presence of a policy. As outlined in recent reviews of physical activity policies in ECEC many have little content or detail and most lack a specific recommendation on how much physical activity (and screen time) children should have per day while at ECEC [19, 34]. Our findings show it is possible to develop setting- and age-specific physical activity and sedentary time recommendations that are meaningful and agreed upon across a range of stakeholders. For example, experts agreed that it was equally viable for the physical activity recommendation to be based either on a standard 6–8-h day in care or as a proportion of the day (i.e., a third of the day spent physically active). Future research is required to understand the impact of the different features of ECEC physical activity policies and implementation strategies on educator physical activity related practices and children’s physical activity behaviour. A first stage of evaluating the impact of the implementation of a new physical activity policy in ECEC should assess change in educator physical activity related practices, and then determine the effect on children’s physical activity levels.

As outlined in a recent Cochrane review of strategies to improve healthy eating, physical activity and obesity prevention policies, practices or programs in ECEC, there are a number of important factors that require consideration for successful implementation of a physical activity policy in ECEC [15]. These are based on the Consolidated Framework for Implementation Research (CFIR) [29] and include overall organisation and individual service director support, parent engagement, resources, education and training for ECEC staff in promoting children’s physical activity, continuous quality improvement and monitoring as well as strategies to overcome key barriers to implementation [15]. Experts in the current study confirmed a number of key CFIR based strategies related to management, supervisors and educators; the ECEC physical environment; communicating with families; and accreditation, monitoring and review, which are necessary for the successful implementation of physical activity policy in ECEC. The findings also identified ‘best bets’ for physical activity policy implementation in ECEC. Experts identified these strategies as both easy to implement and likely to have a high level of influence. These included strategies focussed on daily opportunities for outdoor play; programming for active play and physical activity; breaking up prolonged periods of sedentary time; not withholding physical activity to manage challenging behaviours; promoting awareness of the policy and making it available to all staff, families and visitors; and ensuring staff and children wear appropriate clothing and footwear for physical activity. These strategies are given priority in the physical activity policy template. Focussing on easier to implement strategies that also have a greater influence on educator practice and children’s physical activity behaviour, will provide motivation and encouragement for services to further implement their physical activity policy using other moderate to longer term strategies. This is particularly important given the challenges faced by ECEC providers in implementing new policies while balancing the demands of a crowded curriculum, administrative and reporting requirements and meeting the needs of a successful business [37].

Finally, the experts identified key barriers and enablers to implementing ECEC specific physical activity policies specific to management, supervisors and educators; the ECEC physical environment; and communicating with families. Many of these barriers were consistent with the literature and were common barriers to implementing any type of policy in any organisation i.e., existing workload and competing interests and expectations; lack of training; and costs in time, money and resources [15, 36]. However, some barriers were specific to implementing physical activity policy in ECEC; staff and parent perceptions of risk/injury/illness; insufficient play equipment, lack of indoor and or outdoor space; and weather extremes and have been noted in other studies of ECEC based physical activity interventions [15, 38, 39]. Barriers related to staff and parent perceptions of risk/injury/illness could be addressed through education and training, however barriers related to how supportive the ECEC physical environment is for physical activity may require longer term solutions. Many of the identified barriers to implementing strategies related to the ECEC physical environment and communicating with families. These strategies were identified lowest in terms of ease of implementation in a 1 year period, but would have a strong level of influence on the overall implementation of a physical activity policy in ECEC. Overall, there is little existing evidence of how to overcome implementation barriers related to the ECEC-based physical activity interventions [15].

Not surprisingly, many of the enablers for physical activity policy implementation in ECEC identified by the expert panel were also the opposite to the barriers identified. Two distinct key enablers to implementation were having a clear, concise and easy to follow policy and regulatory requirements for services to have and implement a physical activity policy [15]. The application of implementation science to physical activity intervention research is relatively new, thus further research is required to better understand the setting, context and barriers to implementing physical activity interventions, strategies to overcome these barriers and how to capitalize on implementation enablers [15, 40].

Our findings underscore the importance of the content, coverage, clarity, simplicity, acceptability, and ease of use of physical activity policies developed for ECEC and the need for expert input and also to engage closely with end-users – educators and service directors. Furthermore, having an evidence based clear, concise and easy to follow physical activity policy template for services is necessary but not sufficient. Implementation strategies should be staged, beginning with easy and more influential strategies to enable services to implement their policy (taking into account their existing workload and priorities) and to encourage them to continue with the process. The most powerful way to ensure this happens is for state or national regulations to require all services have a physical activity policy and for it to be implemented. This appears feasible given some regulations currently require services to have policies on other health promoting behaviours such as healthy eating, sun protection and sleep [41].

### Limitations

The round two national survey of experts had less representation from some Australian States and Territories with most based in metropolitan areas. However, there was good representation from different stakeholder groups and a high level of expertise as demonstrated by the high education level and years employed in the field. Invitation to take part in the round two survey of experts was based on an identified list of experts and stakeholders, however there was evidence of the survey link being forwarded to others who may have not been considered an expert. In addition, not all (75%) of participants completed both the second and third round surveys which may have resulted in the exclusion of some experts views on the level of influence of strategies to implement physical activity policy in ECEC and the key barriers and enablers to these implementation strategies. As per the Delphi methodology, the survey data collected were subjective and based on expertise and the knowledge and opinion of participants.

## Conclusions

The Delphi process enabled the development of a specific evidence-based physical activity policy template for ECEC and the endorsement of strategies to support its successful implementation. This research is timely given a large proportion of children who attend ECEC are insufficiently active and many ECEC services do not have a comprehensive physical activity policy to guide the amount and type of physical activity children are involved in whilst attending ECEC. The physical activity policy template will be implemented and evaluated to determine its impact on educator physical activity-related practices and children’s physical activity behaviour. This research will provide further weight to the call for state and national ECEC regulations to ensure that services have physical activity policies and procedures in place and support quality implementation.

## Data Availability

The datasets used and analysed during the current study are available from the corresponding author on reasonable request. A copy of the ECEC specific physical activity policy template is available from the first author by request.

## Abbreviations

ECEC: Early Childhood Education and Care
PLAYCE: PLAY Spaces and Environments for Children’s Physical Activity project
